# A fast and accurate zebra finch syllable detector

**DOI:** 10.1371/journal.pone.0181992

**Published:** 2017-07-28

**Authors:** Ben Pearre, L. Nathan Perkins, Jeffrey E. Markowitz, Timothy J. Gardner

**Affiliations:** 1 Department of Biology, Boston University, Boston, Massachusetts, United States of America; 2 Department of Neurobiology, Harvard Medical School, Boston, Massachusetts, United States of America; Texas Christian University, UNITED STATES

## Abstract

The song of the adult male zebra finch is strikingly stereotyped. Efforts to understand motor output, pattern generation, and learning have taken advantage of this consistency by investigating the bird’s ability to modify specific parts of song under external cues, and by examining timing relationships between neural activity and vocal output. Such experiments require that precise moments during song be identified in real time as the bird sings. Various syllable-detection methods exist, but many require special hardware, software, and know-how, and details on their implementation and performance are scarce. We present an accurate, versatile, and fast syllable detector that can control hardware at precisely timed moments during zebra finch song. Many moments during song can be isolated and detected with false negative and false positive rates well under 1% and 0.005% respectively. The detector can run on a stock Mac Mini with triggering delay of less than a millisecond and a jitter of *σ* ≈ 2 milliseconds.

## 1 Introduction

The adult zebra finch (*Taeniopygia guttata*) sings a song made up of 2–6 syllables, with longer songs taking on the order of a second. The song may be repeated hundreds of times per day, and is almost identical each time. Several brain areas reflect this consistency in highly stereotyped neural firing patterns, which makes the zebra finch one of the most popular models for the study of the neural basis of learning, audition, and control.

If precise moments in song can reliably be detected quickly enough to trigger other apparatus during singing, then this consistency of behaviour allows a variety of experiments. A common area of study with song-triggered experiments is the anterior forebrain pathway (AFP), a homologue of mammalian basal ganglia consisting of a few distinct brain areas concerned with the learning and production of song. For example, stimulation of the lateral magnocellular nucleus of the anterior nidopallium (LMAN)—the output nucleus of the AFP—at precisely timed moments during song showed that this area controls specific variables in song output [1]. Song-synchronised stimulation of LMAN and the high vocal centre (HVC) in one hemisphere or the other showed that control of song rapidly switches between hemispheres [2]. Feedback experiments have shown that Field L and the caudolateral mesopallium may hold a representation of song against which auditory signals are compared [3]. The disruption by white noise of renditions of a syllable that were slightly above (or below) the syllable’s average pitch showed that the apparently random natural variability in songbird motor output is used to drive change in the song [4], and the AFP produces a corrective signal to bias song away from those disruptions [5]. The song change is isolated to within roughly 10 milliseconds (ms) of the stimulus, and the shape of the learned response can be predicted by a simple mechanism [6]. The AFP transfers the error signal to the robust nucleus of the arcopallium (RA) using NMDA-receptor–mediated glutamatergic transmission [7]. The course of song recovery after applying such a pitch-shift paradigm showed that the caudal medial nidopallium is implicated in memorising or recalling a recent song target, but in neither auditory processing nor directed motor learning [8].

Despite the power and versatility of vocal feedback experiments, there is no standard syllable detector. Desiderata for such a detector include:

**Accuracy:** How often does the system produce false positives or false negatives?
**Latency:** The average delay between the target syllable being sung and the detection.
**Jitter:** The amount that latency changes from instance to instance of song. Our measure of jitter is the standard deviation of latency.
**Versatility:** Is detection possible at “difficult” syllables?
**Ease of use:** How automated is the process of programming a detector?
**Cost:** What are the hardware and software requirements?

A variety of syllable-triggering systems have been used, but few have been documented or characterised in detail. In 1999, detection was achieved by a group of IIR filters with hand-tuned logical operators [9]. The system had a latency of 50 or 100 ms, and accuracy and jitter were not reported. As access to computational resources has improved, approaches have changed: in 2009, hand-tuned filters were implemented on a Tucker-Davis Technologies digital signal processor, bringing latency down to around 4 ms [5]. But as with other filter-bank techniques, it is not strictly a syllable detector but rather a pitch and timbre detector—it cannot identify a frequency sweep, or distinguish a short chirp from a long one—and thus requires careful selection of target syllables. Furthermore, the method is neither inexpensive nor, based on our experience with a similar technique, accurate. 2009 saw the application of a neural network to a spectral image of song [3]. They reported a jitter of 4.3 ms, but further implementation and performance details are not available. In 2011, stable portions of syllables were matched to spectral templates in 8-ms segments [7]. This detector achieved a jitter of 4.5 ms, and false-negative and false-positive rates of up to 2% and 4% respectively. Hardware requirements and ease of use were not reported. In 2013, spectral images of template syllables were compared to song using a correlation coefficient [10]. With a fast desktop (Intel i7 six-core) running Linux and equipped with a National Instruments data acquisition card, it boasts a hardware-only (not accounting for the time taken to compute a match with a syllable) latency and jitter of just a few microseconds, and the detection computation they use should not much increase that. They reported false-negative rates around 4% and 7% for zebra finches and Bengalese finches respectively, measured on a small dataset. In much other work, a syllable detector is alluded to, but not described.

We developed a standalone detector that learns to match moments in the song using a neural network applied to the song spectrogram, and outputs a TTL pulse (a brief 5-volt pulse) at the chosen moment. The approach consists of three steps:

1. Record and align a corpus of training songs. The technique has been published in [11]. As few as 200 songs can yield acceptable results, but here we standardise on 1000-song training sets.
2. Choose one or more instants in the song that should create a trigger event, and train a neural network to recognise them. This step is carried out offline. While any neural network software would produce similar results, we used MATLAB 2015b’s neural network toolbox.
3. Once trained and saved, the neural network is used by a realtime detection programme that listens to an audio signal and indicates detection of the target syllables via a TTL pulse. We present three detection implementations, in MATLAB, LabVIEW, and Swift, that trade off hardware requirements, ease of maintenance, and performance.

This method makes the following contributions:

- Fast: sub-millisecond latencies, with jitter around 2 ms.
- Accurate: false negative rates under 1% and false positive rates under 0.005% for a variety of syllables.
- State-of-the-art performance with default parameters.
- Requires almost no programming experience.
- Runs on inexpensive hardware.
- Described in detail here, with reference implementations provided and benchmarked.

## 2 Materials and methods

### 2.1 Training a detector

We begin with a few hundred recordings of a given bird’s song, as well as calls, cage noise, and other non-song audio data. Male zebra finch song is most highly stereotyped when a female is present (“directed song”) and slightly more variable otherwise (“undirected”); we train and test on undirected song since this is both more commonly studied and more challenging. Recordings were made inside a sound-isolating chamber in which was mounted a recording microphone (Audio-Technica AT803), using methods similar to those described in [12], Chapter 2. The songs were time-aligned as described in [11].

We rely on two circular buffers:

**Audio buffer:** This contains the most recent audio samples, and is of the length required to compute the Fast Fourier Transform (FFT)—usually 256 samples.
**FFT buffer:** The results of each new FFT are placed here. It contains the *n*_fft_ most recent FFTs, which will serve as inputs to the neural network (described below).

Audio is recorded at some sample rate 1/*t*_sample_ (for example, 44.1 kHz), and new data are appended to the circular audio buffer.

**Fig 1:**
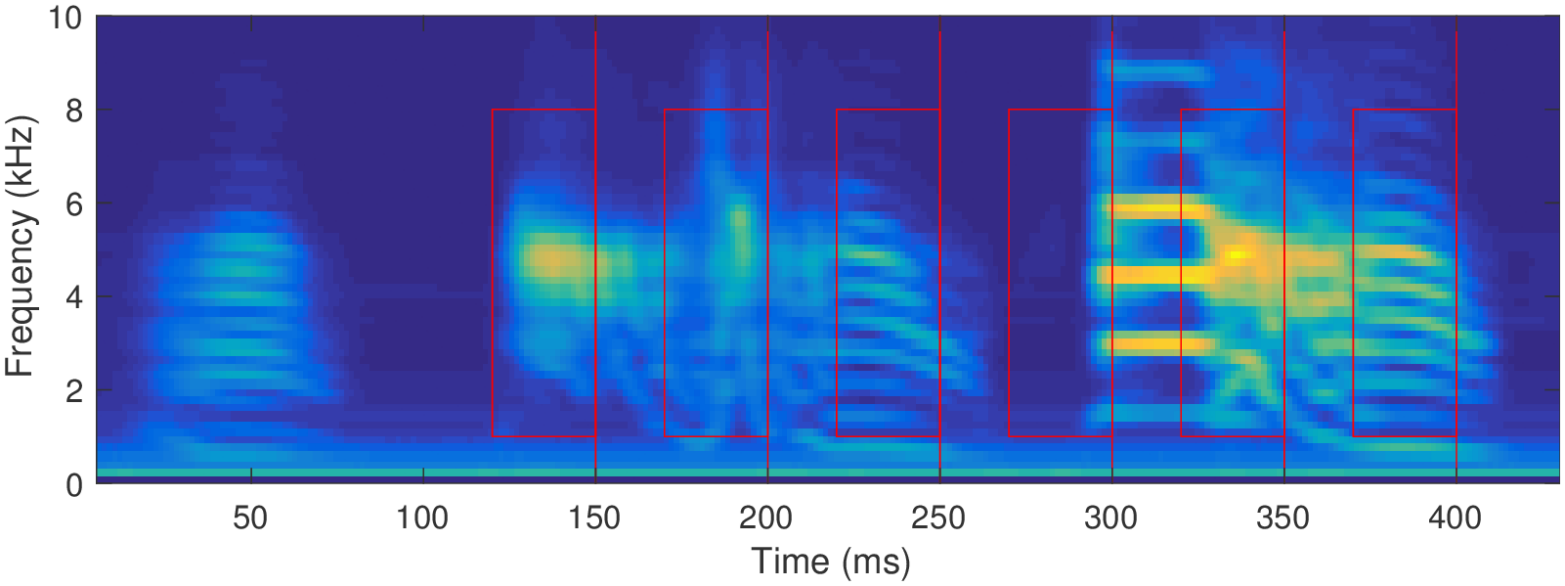
The spectrogram of the song of the bird “lny64”, used as an example throughout this paper. This image was made by superposing the spectra of our 2818 aligned songs. Our example detection points, $t_{1}^{*}\ldots t_{6}^{*}$, are shown as red lines, with example recognition regions of 30 ms × 1–8 kHz marked as rectangles.

The spectrogram is computed at regular intervals—the useful range seems to be roughly 1–5 milliseconds, which we refer to as the frame interval *t*_fft_. At each frame, a spectrum is computed from the most recent 256 audio samples in the audio buffer, and the result is appended to the FFT buffer. For example, if *t*_fft_ = 1 ms and the recording sample rate 1/*t*_sample_ = 40 kHz, then in order to compute a new FFT, 40000 ⋅ 0.001 = 40 new audio samples must be appended to the audio buffer, the FFT is computed using the most recent 256 samples in that buffer, and the result is appended to the FFT buffer. Over time, the successive spectra will look something like Fig 1.

This results in time being discretised into chunks of length *t*_fft_. Because each Fourier transform computation contains a small number *n*_*fft*_ of new audio samples (in the example above, 40 new samples and 256 − 40 that have already been examined), we tried implementing the Sliding Discrete Fourier transform (SDFT) [13]. This allows *t*_fft_ = *t*_sample_. Practically, the operating system retrieves new audio samples from the hardware several at a time, so the full benefit of the SDFT is difficult to see in practice. Furthermore, we found that FFT implementations are sufficiently highly optimised that discretising time into chunks of *t*_fft_ as we have done produced similar results with simpler software.

When using consumer audio hardware that operates best at 44.1 kHz, the requested frame interval may not line up with the sample rate, so the actual frame interval may be different from the intended. For example, at 44.1 kHz a 1-ms frame interval requires a new FFT every 44.1 samples. This must be rounded to 44 samples, resulting in *t*_fft_ = ⌊44.1⌉/44.1 ≈ 0.9977 ms.

One or more target moments during the song must be chosen. Our interface presents the time-aligned spectrogram averaged over training songs, and requires manual input of the target times, t*. Then we assemble the training set from the song data, train the network, compute optimal output unit thresholds, and save the network object and an audio test file.

#### 2.1.1 Recognition region

The neural network’s inputs are the FFT values from a rectangular region of the spectrogram covering a predefined range of frequency values *F* (for example, 1–8 kHz) at some number of the most recent frames *n*_fft_. Any time *t* falls within frame *τ*(*t*), and *t* − *t*_fft_ falls within the previous frame, so the recognition region that the neural network receives as input consists of the spectrogram values over *F* at *τ*(*t*) and those from the contiguous set of recent frames: *T* = { *τ*(*t*), *τ*(*t* − *t*_fft_), *τ*(*t* − 2*t*_fft_)… *τ*(*t* − *n*_fft_ *t*_fft_)}. Time windows of 30–50 ms—the latter will yield *n*_fft_ = |*T*| = ⌊50 ms/*t*_fft_⌉ frames—of frequencies spanning 1–8 kHz generally work well.

Six examples of chosen target moments in the song, with recognition regions *F* = 1–8 kHz and *T* = 30 ms, are shown in Fig 1.

#### 2.1.2 Building the training set

The training set is created in a fashion typical for neural networks: at each time frame *t* the rectangular |*F*| × |*T*| recognition region in the spectrogram as of time *t* is reshaped into a vector *ξ*_*t*_, which will have length |*F*||*T*| and contain the spectra in *F* taken at all of the times in the set *T*: from *τ*(*t* − *n*_fft_*t*_fft_) to *τ*(*t*). These vectors are placed into a training matrix, *Ξ*, such that each column *ξ*_*t*_ holds the contents of the recognition region—containing multiple frames from the spectrogram—as of one value of *t*.

Training targets *y*_*t*_ are vectors with one element for each desired detection syllable. That element is, roughly, 1 if the input vector matches the target syllable (*t* = t*), 0 otherwise, for each target syllable (of which there may be any number, although they increase training time, and in our implementations the number of distinct output pulses is constrained by hardware). Since the song alignment may not be perfect, and due to sample aliasing, a strict binary target may ask the network to learn that, of two practically identical frames, one should be a match and the other not. Thus it is preferable to spread the target in time, such that at the target moment it is 1, and at neighbouring moments it is nonzero. We found that a Gaussian smoothing kernel around the target time with a standard deviation of 2 ms serves well.

With inputs well outside the space on which a neural network has been trained, its outputs will be essentially random. In order to reduce the false positive rate it is necessary to provide negative training examples that include silence, cage noise, wing flapping, non-song vocalisations, and perhaps songs from other birds. Although it will depend on the makeup of the non-song data, we have found that training with as low as a 1:1 ratio of non-song to song—or roughly 10 minutes of non-song audio—yields excellent results on most birds.

#### 2.1.3 Normalisation

In order to present consistent and meaningful inputs to the neural network and to maximise the effectiveness of the neural network’s training algorithm, we normalise the incoming data stream so that changes in the song structure of the sound are emphasised over changes in volume.

The first normalisation step is designed to eliminate differences in amplitude due to changes in the bird’s location and other variations in recording. Each recognition region vector *ξ*_*t*_—during training, each *column* of the training matrix Ξ—is normalised using MATLAB’s zscore function ${\hat{\xi }}_{t}=\left(\xi_{t}-\mu_{\xi_{t}}\right)/\sigma_{\xi_{t}}$, so that the content of each window has mean $\mu_{{\hat{\xi }}_{t}}=0$ and standard deviation $\sigma_{{\hat{\xi }}_{t}}=1$.

The second step is designed to ensure that the neural network’s inputs have a range and distribution for which the training function can easily converge. Each element *i* of $\hat{\xi }$ is normalised across the entire training set—each *row* of Ξ—in the same way: ${\overset{ˇ}{\xi }}^{i}=\left({\hat{\xi }}^{i}-\mu_{{\hat{\xi }}^{i}}\right)/\sigma_{{\hat{\xi }}^{i}}$, so that the values of that point across the whole training set have mean $\mu_{{\overset{ˇ}{\xi }}^{i}}=0$ and standard deviation $\sigma_{{\overset{ˇ}{\xi }}^{i}}=1$. This is accomplished during training by setting MATLAB’s neural network toolbox normalisation scheme to mapstd, and the scaling transform is saved as part of the network object used by the realtime detector so that it may be applied to unseen data at runtime.

These two normalisation steps provide a set of inputs that are more robust to outliers and less likely to produce false positives during silence than other normalisation schemes, such as linear or L_2_ normalisation.

#### 2.1.4 Neural networks

While any learned classifier might suffice, we chose a two-layer feedforward neural network. In brief, our network takes an input vector *ξ*_*t*_—as described above—and produces an output vector *y*_*t*_, and when any element of *y*_*t*_ is above a threshold (described below), the detector reports a detection event. The network uses two matrices of weights, *W*_0_ and *W*_1_, and two vectors of biases, *b*_0_ and *b*_1_. The first takes the input *ξ*_*t*_ to an intermediate stage—the “hidden layer” vector. To each element of this vector is applied a nonlinear squashing function such as tanh, and then the second weight matrix *W*_1_ is applied. This produces output *y*_*t*_:

$$\begin{array}{c} y_{t}=W_{1}\tanh\left(W_{0}\xi_{t}+b_{0}\right)+b_{1} \end{array}$$

During the network’s training phase, the elements of the matrices and bias vectors are learned by back-propagation of errors over a training set. A more detailed explanation of neural networks may be found in [14].

Essentially, after training, the network is available in the form of the two matrices *W*_0_ and *W*_1_ and the two vectors *b*_0_ and *b*_1_, and running the network consists of two matrix multiplications, two vector additions and the application of the squashing function.

#### 2.1.5 Training the network

The network is trained using MATLAB’s neural network toolbox, with Scaled Conjugate Gradient (trainscg). We tried a variety of feedforward neural network geometries, from simple 1-layer perceptrons to geometries with many hidden nodes, as well as autoencoders. Perhaps surprisingly, even the former yields excellent results on many syllables, but a 2-layer perceptron with a very small hidden layer—with a unit count 2-4 times the number of target syllables—was a good compromise between accuracy and training speed. For more variable songs, deep structure-preserving networks may be more appropriate, but they are slow to train and unnecessary for zebra finch song.

#### 2.1.6 Computing optimal output thresholds

After the network is trained, outputs of the network for any input are now available, and will be in (or, due to noisy inputs and imperfect training, close to) the interval (0, 1). We must choose a threshold above which the output is considered a positive detection. Finding the optimal threshold requires two choices. The first is the relative cost of false negatives to false positives, *C*_*n*_. The second is the acceptable time interval: if the true event occurs at time *t*, and the detector triggers at any time *t* ± Δ*t*, then it is considered a correct detection. Then the optimal detection threshold is the number that minimises [false positives] + *C*_*n*_ ⋅ [false negatives] over the training set, using the definitions of false positives and negatives given in Section 2.3.1. Since large portions of the cost function are flat, we use a brute-force linear search, which requires fractions of a second. For the results presented here, we have used Δ*t* = 10 ms, and arbitrarily set *C*_*n*_ = 1.

#### 2.1.7 De-bouncing

During runtime, the network may produce above-threshold responses to nearby frames. Thus, after the first response, subsequent responses are suppressed for 100 ms. However, for the accuracy measurements presented here, we used the un-de-bounced network response.

#### 2.1.8 Our parameter choices

We used an FFT of size 256; a Hamming window; and chose a target spectrogram frame interval of *t*_fft_ = 1.5 milliseconds, resulting in a true frame interval of *t*_fft_ = ⌊1.5 ⋅ 44.1⌉/44.1 ≈ 1.4966 ms. We set the network’s input space to 50 ms long, and to span frequencies from 1–8 kHz, which contains the fundamentals and several overtones of most zebra finch vocalisations.

We found these parameters to work well across a variety of target syllables, but various other parameter sets yield results similar to those presented here. Some of the parameters trade off detection accuracy or temporal precision vs. training time. For example, decreasing the frame interval generally decreases both latency and jitter, but also increases training time. Sometimes the effects are syllable-specific: for example, using a 30-ms time window rather than 50 ms speeds training while usually having a minimal effect on detector performance (as is the case, for example, for all of the detection points for the bird “lny64”), but occasionally a syllable will be seen for which extending the window to 80 ms is helpful.

### 2.2 Realtime detection

The architecture of the realtime detector requires that the most recent *n*_fft_ spectrograms be fed to the neural network every frame interval. Audio samples from the microphone are appended to the circular audio buffer. Every *t*_fft_ seconds a new spectrogram is calculated by applying the Hamming window to the contents of the buffer, performing an FFT, and extracting the power. Outputs of the spectrogram from the target frequency band are appended to the circular FFT buffer. The spectrograms are sent to a static implementation of the previously trained neural network.

We tested three implementations of the realtime detector. For all of these tests, we ran the detector processes under the operating systems’ default schedulers and process priorities, running typical operating system daemon processes but no loads from user processes. The computers had ample memory resources.

#### 2.2.1 Swift

This detector uses the Swift programming language and Core Audio interface included in Apple’s Mac OS X operating systems.

The Core Audio frameworks provide an adjustable hardware buffer size for reading from and writing to audio hardware (different from our two circular buffers). Tuning this buffer size provides a tradeoff between the jitter in the detection and the processor usage needed to run the detector. We used buffer sizes ranging from 8 samples (0.18 ms at 44.1 kHz) to 32 samples (0.7 ms at 44.1 kHz) depending on the frame size used by the detector.

Vector operations—applying the Hamming window, the FFT, input normalisation, matrix multiplication, and the neural network’s transfer functions—are performed using the Accelerate framework (vDSP and vecLib), which use modern vector-oriented processor instructions to perform calculations.

When the neural network detects a match, it instructs the computer to generate a TTL pulse that can be used to trigger downstream hardware. This pulse can be either written to the computer’s audio output buffer (again, in 8- to 32-sample chunks) or sent to a microcontroller (Arduino) via a USB serial interface. Sending the trigger pulse via the serial interface and microcontroller is noticeably faster (2.2 ms lower latency), likely due to the fact that the audio buffer goes through hardware mixing and filtering prior to output.

The above code can be run on multiple channels of audio on consumer hardware (such as a 2014 Mac Mini) with little impact on CPU usage (<15%). Depending on the experimental needs, latency can potentially be further decreased (at the expense of processor usage) by adjusting the audio buffer sizes.

We ran the Swift detector on a Late 2014 Mac Mini with a Intel Core i5 processor at 2.6GHz with 16 gigabytes of RAM, running Mac OS X 10.11.

#### 2.2.2 LabVIEW

This implementation requires special software and hardware: LabVIEW from National Instruments—we used 2014 service pack 1—and a data acquisition card—we use the National Instruments PCI-6251 card on a PC with an Intel Core i5-4590 processor at 3.7GHz (a relatively low-end machine) with 24 gigabytes of RAM, running Microsoft Windows 8.1 Pro and Windows 10.

This implementation has several drawbacks: it requires expensive hardware and software from National Instruments (a data acquisition card and LabVIEW), Windows (we use hardware features of LabVIEW that are unavailable on MacOS or Linux), and due to the programming language it is difficult to modify and debug—indeed, a persistent bug in our implementation currently renders it substantially less accurate than the other detector implementations on some syllables. However, our test configuration proved itself capable of excellent performance, and further gains should be possible if the implementation were retargeted onto field-programmable gate array (FPGA) hardware—which would have the additional benefit of providing deterministic “hard realtime” guarantees—or just run on a faster desktop system.

#### 2.2.3 MATLAB

This detector uses the built-in audio input and output hardware on a compatible computer. We tested on a 2014 Mac Mini (the same machine used for the Swift detector described above) and 2015 Mac Pro. The Mac Pro does not have an audio input jack, so input was through an M-Audio MobilePre external USB audio interface. Despite the faster processor, the latter system did not achieve higher performance than the former, due to USB data transfer overhead.

Because of how MATLAB’s DSP toolbox interfaces with audio hardware, there is a double buffer both reading from and writing to audio hardware. As a result, much of the code focuses on a lightweight audio hardware interface, in order to have the smallest achievable audio buffer. To help achieve this, the MATLAB implementation spreads data acquisition and processing across two threads, due to the higher computational overhead of the interpreted programming language.

The most versatile implementation, MATLAB runs on a variety of hardware and operating systems, and is perhaps the easiest to modify. While it did not perform as well as the other implementations, the convenience may outweigh the timing performance penalty for some experiments. Key to minimising jitter is the size of the audio buffer: on a 2014 Mac Mini running MATLAB 2015b the smallest buffer size that did not result in read overruns was about 4 ms.

As with the Swift detector, it is also possible to modify the MATLAB implementation to generate the TTL pulse from a microcontroller (Arduino) controlled via a serial over USB interface. This eliminates the double buffer required when sending triggers via the audio interface, reducing latency by close to half.

### 2.3 Quantification

We measure accuracy and characterise timing for 6 randomly chosen points in one bird’s song (lny64), and present accuracy results for seven more birds (the detector’s timing does not appreciably vary across birds). The results presented here are representative of real-world performance in feedback experiments on many birds in our lab.

Data consisted of extracted, aligned songs, and additional song-length samples of non-song (cage noise, calls, other birds’ song). These were divided into training and test sets. For all birds, we used 1000 songs and 1000 non-song samples for training. The test set consisted of the remaining songs and an equal number of non-songs. Table 1 lists the test dataset sizes and target detection points. For lny64, we arbitrarily chose six linearly spaced target times. For each of the other birds, we eyeballed one promising-looking detection point.

**Table 1:** For each bird, detectors were trained for the specified timepoints, using 1000 songs and 1000 non-song samples of the same length. The test set consisted of the remaining songs and an equal number of non-songs.

Bird	Test songs = Test non-songs	Target time (ms)
lny64	1818	150:50:400
lny46	1000	195
lny42	1245	380
lny4rb	1000	250
lr12	2000	180
lr13	2000	300
lr28	3000	140
lr77	2000	320

The MATLAB neural network toolbox further divides our “training” set into internal training, validation, and test sets. Because we do our own testing on a distinct data set, we instructed the toolbox to use 80% of its input set for training, and the remaining 20% for validation, with no hold-out test data.

Because the data set consists of temporally aligned songs, the moment at which detection should occur during each song sample is available (albeit with minor variations due to the alignment process used [11]). The detector can be checked by comparing its response to the recorded training songs against these “canonical” detection events. To this end, besides the trained network object, our learning code produces an audio file consisting of all of the training data on the left audio channel and a delta function at each aligned moment of target syllable presentation on the right channel. Thus, when played on any audio player, the left channel may be provided as input to the detector, and the the detector’s output pulses may be compared against the “ground truth” given by the canonical detection event provided by the right channel.

#### 2.3.1 Accuracy

We define the accuracy of the network based on its classification performance per frame. In order to avoid the apparent problem of every non-detected non-matching frame counting as a true negative, we also tried defining accuracy on a per-song basis, such that a song-length sample without the target syllable counted as a single true negative. Computing the optimal output thresholds on a per-frame rather than a per-song basis resulted in higher thresholds and thus a slight reduction in both the false-positive and true-positive rates (reversible by increasing *C*_*n*_), while also providing a valid definition of the false-positive rate for data streams that had not been segmented into song-sized chunks and thus allowing easy interpretation of false-positive rates from calls, cage noise, etc.

The accuracy as defined above is used for computing the optimal thresholds above which the network’s output should be interpreted as a match on the training data as described in Section 2.1.6, for evaluation of the detectors on the training songs, and while live.

#### 2.3.2 Timing

We evaluate the time taken from the complete presentation of each instance of the target syllable to the firing of the detector’s TTL pulse. For example, if the target trigger point is at 200 ms with respect to the corpus of aligned songs, and if the detection region is 30 ms long, then when the detector has seen the region from 170–200 ms, it should recognise the syllable. If for a given presentation of the song the detector fires at 203 ms, we take the latency on that trial to be 3 ms.

While playing the audio test file from any audio playback device, we used the TTL output from the ground-truth channel of the audio output as the trigger pulse for an oscilloscope, and compared it to the TTL pulse produced by the detector, which sees only the birdsong channel of the audio file. For this purpose we used a pulse generator (Philips PM 5715, with a listed latency of 50 ns, jitter of ≤ 0.1% or 50 ps, whichever is greater) to widen the detector’s output spike to a number much larger than the jitter (∼100 ms). This obviates pulse length variability in the output device by essentially discarding the falling edge of the output pulse. The oscilloscope is then set to averaging mode (128-trigger average) in order to collect timing data. The canonical signal is the trigger at *t* = 0, and the average of the detector’s detection events will be seen as a low-to-high transition with form approximating the cumulative probability distribution function (CDF) of the detector’s output in response to the chosen song event, with individual detection events visible as steps in this curve.

We define latency as the time between the training target (during our tests, this is indicated by the canonical signal in the test file) and the corresponding detection event. It is a helpful number, but not a critical one; due to the stereotyped song of the zebra finch, a detector with high but constant latency can often be trained to trigger at a point somewhat before the true moment of interest (for example, if triggering should occur at the very beginning of a syllable, the detector may be trained to recognise the previous syllable and the gap thereafter). Usually the variability in latency is the more important number. We define jitter as the standard deviation of detection latency, measured over the test songs.

When measuring timing, it is useful to compare against a theoretical optimal, in order to control for any imprecision in the song extraction and alignment of our real data inherent in the method we use (described in [11]). We propose two measures thereof:

First we test the “ideal” latency and jitter in the time shift used in calculating new columns of the spectrogram. By passing a recorded audio sequence into the detector offline, and assuming no input or output latency, we compare how many additional audio samples beyond the syllable are needed before the spectrogram can match the inputs needed to trigger the neural network. This latency reflects the FFT size used for calculating the spectrogram, the FFT time shift between columns in the spectrogram, and the width of the Gaussian smoothing kernel applied to the ground truth data when training the neural network, but ignores computation times, audio buffers, and other operating system overhead.

Next we use a “*δ*-syllable” consisting of a *δ* function in the time domain, and train the network to trigger 5 ms after this pulse. This song is fed into the live detector. The results for this measurement show the latency and jitter inherent in the complete detector including audio read and write buffers, classifier computation, and FFT time shift aliasing, but excluding imprecision in the song alignment as well as detection timing effects due to differences between instances of the bird’s song.

Finally, we measure latency on real bird song aligned as in [11]. This extracts and aligns the centres of the sampled songs, and the canonical signal is given with respect to that alignment. These timing measurements reflect not only detector performance, but also the variability of the bird’s song.

### 2.4 Ethics

All procedures were approved by the Institutional Animal Care and Use Committee of Boston University (protocol number 14-029).

## 3 Results

Fig 1 shows a typical song. The image is produced by averaging the aligned spectrograms for the bird lny64. Six target trigger points are shown for this song, at 150, 200, 250, 300, 350, and 400 milliseconds after the beginning of the aligned samples, as indicated by the red lines. In discussion, we will refer to these six as $t_{1}^{*}\ldots t_{6}^{*}$ respectively. To avoid overlap in the graphic, the rectangles show a shorter recognition window than our standard: here 30 ms, but still spanning 1–8 kHz, for each target time in the song.

The first two triggers, $t_{1}^{*}$ at 150 ms and $t_{2}^{*}$ at 200 ms, are far from pure tones, but are closer to bandpass-filtered white noise. They would be difficult for a harmonic-stack filter bank to detect, and especially to pinpoint in time. $t_{3}^{*}$ (250 ms) and $t_{6}^{*}$ (400 ms) are rich in overtones and contain downward slides with changing timbres. $t_{4}^{*}$ (300 ms) occurs near the beginning of a more typical harmonic stack amenable to a variety of recognition techniques, although consistently detecting a point partway through the syllable demands a detector that can use multiple time steps. $t_{5}^{*}$ (350 ms) shows a steady pitch followed by a complex changing tone structure.

**Fig 2:**
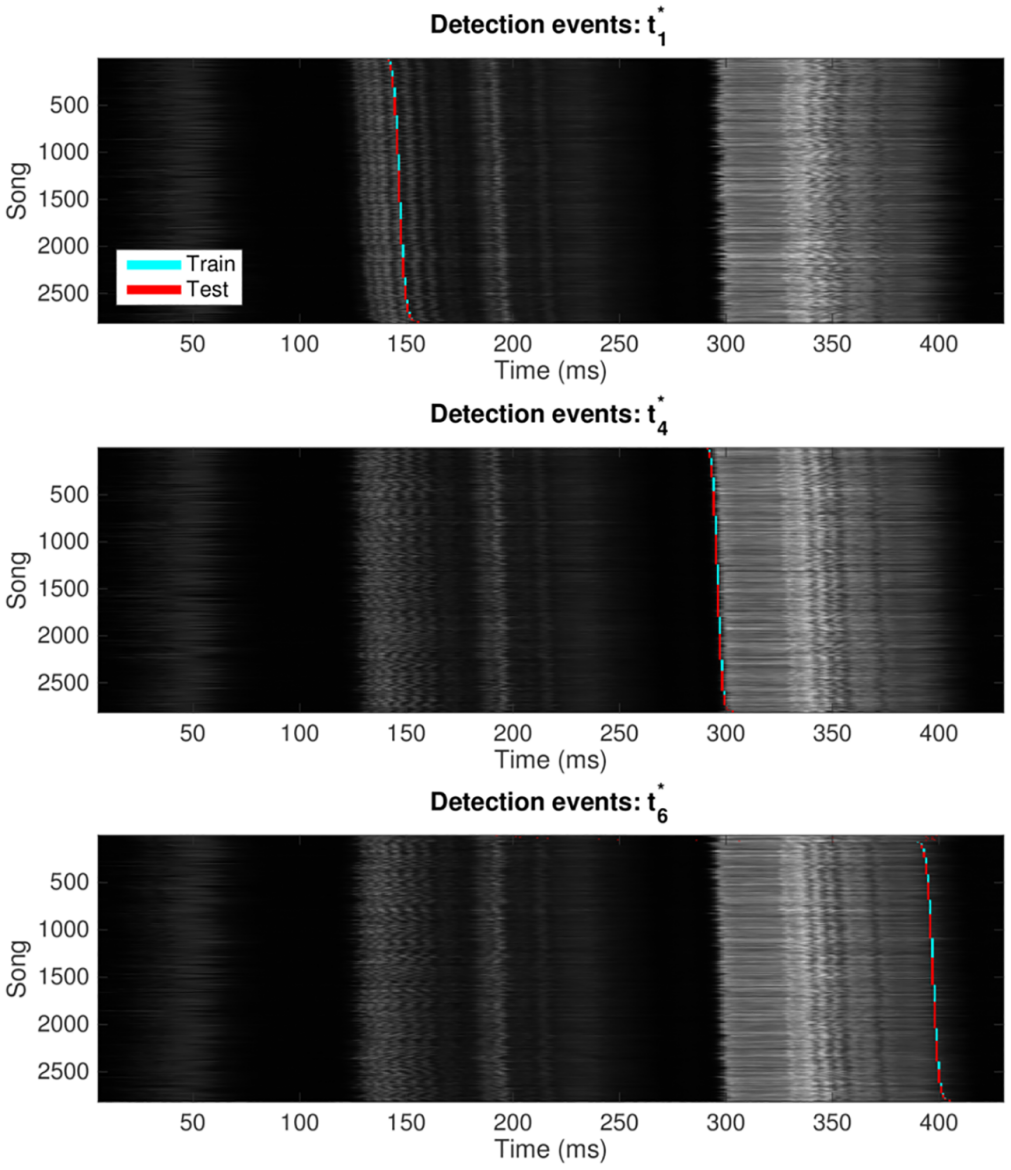
Each plot shows one network output unit’s responses to all 2818 presentations of lny64’s song shown in Fig 1. We show only the syllables $t_{1}^{*}$, $t_{4}^{*}$, and $t_{6}^{*}$, and we do not show the non-response to presentation of non-song. The horizontal axis is time relative to the beginning of the aligned song, and the vertical axis is an index for the 2818 individual song presentations. The grey shading shows the audio amplitude of song Y at time T. Detection events on training songs are shown in cyan, with detections of unseen test songs in red. To provide an intuition of intra-song variability, songs have been stably sorted by the time of detection events; thus, each of the three detection graphs shows the songs in a different order.

An example of the detector’s output for three of these syllables is shown in Fig 2. The total audio energy of each song is shown as a single grayscale row. By sorting according to time of each syllable’s detection, the rest of the song is shown in the context of the timing for that moment. This gives an intuition of the timing variability within each song and across songs (which is responsible for a small amount of our measured jitter, but note that few of the songs are significantly time-shifted).

### 3.1 Accuracy

We allowed our training software to allocate our default of 4 hidden units per syllable, and computed a new FFT every 1.5 ms. Because our timing test files are designed for our stereo playback software, allowing only one channel for the ground-truth pulse, we trained one detector for each syllable for the following results. In order to eliminate the large number of variables involved in microphone, cage, playback and re-digitising, we evaluated the neural network’s accuracy directly on the digitised recording. When training and runtime data are gathered on the same hardware setup, this is the digital signal that the detector will see.

**Fig 3:**
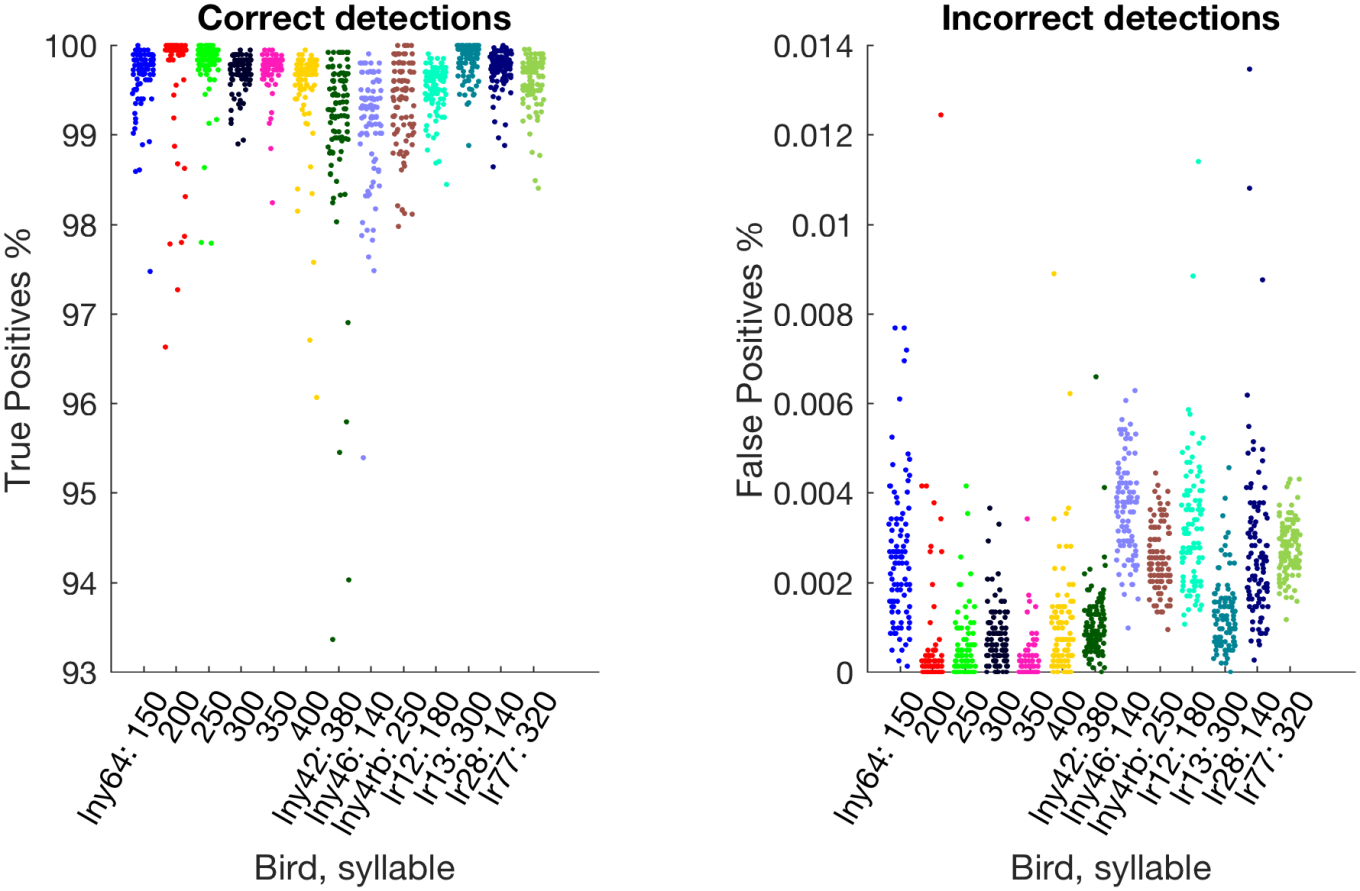
Accuracy variability over 100 different training runs for each of the test detection points. Each dot shows the test-set accuracy for an independently trained detector. Because the horizontal positions have been randomised slightly so as not to occlude same-valued measurements, test syllable is also indicated by colour. The means are given in Table 2.

Accuracies are shown in Fig 3 (accuracies for the synthetic *δ*-function songs were always 100%). For each test syllable we trained 100 different networks, which differ from each other both in the random initialisation of network weights and in the random subset of the song-and-nonsong corpus that was used for training. Each network’s accuracy is shown as a single point in each chart in Fig 3, and the means are shown in Table 2. Evidently some syllables are easier to identify reliably than others (for example, for lny64, $t_{3}^{*}$ (250 ms) and $t_{6}^{*}$ (400 ms) are occasionally confused due to their similar appearance, as could be guessed from Fig 1); thus, if the experimental design permits, it can be worthwhile to choose an easily identifiable syllable. Furthermore, as can be seen from Fig 3, for difficult syllables the training process occasionally yields a network that performs unusually poorly. It is easy to spot these networks by their performance either as measured as part of the training process or on the test audio file, perhaps taking the best of a few training runs.

**Table 2:** Mean values for the detection accuracies shown in Fig 3.

Bird	Target time (ms)	% True positives	% False positives
lny64	150 $\left[t_{1}^{*}\right]$	99.66	0.0026
	200 $\left[t_{2}^{*}\right]$	99.75	0.00052
	250 $\left[t_{3}^{*}\right]$	99.79	0.00051
	300 $\left[t_{4}^{*}\right]$	99.70	0.00077
	350 $\left[t_{5}^{*}\right]$	99.73	0.00022
	400 $\left[t_{6}^{*}\right]$	99.52	0.0010
lny42	380	99.09	0.0011
lny46	140	99.11	0.0036
lny4rb	250	99.37	0.0026
lr12	180	99.52	0.0033
lr13	300	99.83	0.0013
lr28	140	99.73	0.0028
lr77	320	99.58	0.0028

We repeated the above experiment using only 2 hidden units per syllable. Training under this condition is much faster, but the mean true positive rate per syllable decreases by an average of 18% across our test syllables, and the mean false positive rate increases by roughly 23%.

Changing the FFT interval to 2 ms significantly reduces the computational requirement during learning. For most syllables, results were identical, but some syllables are slightly easier to detect and some slightly more difficult, with no significant difference on average.

### 3.2 Timing

We evaluate variability in timing performance across three variables: FFT frame interval; syllable choice; and detector implementation.

#### 3.2.1 FFT frame interval

**Fig 4:**
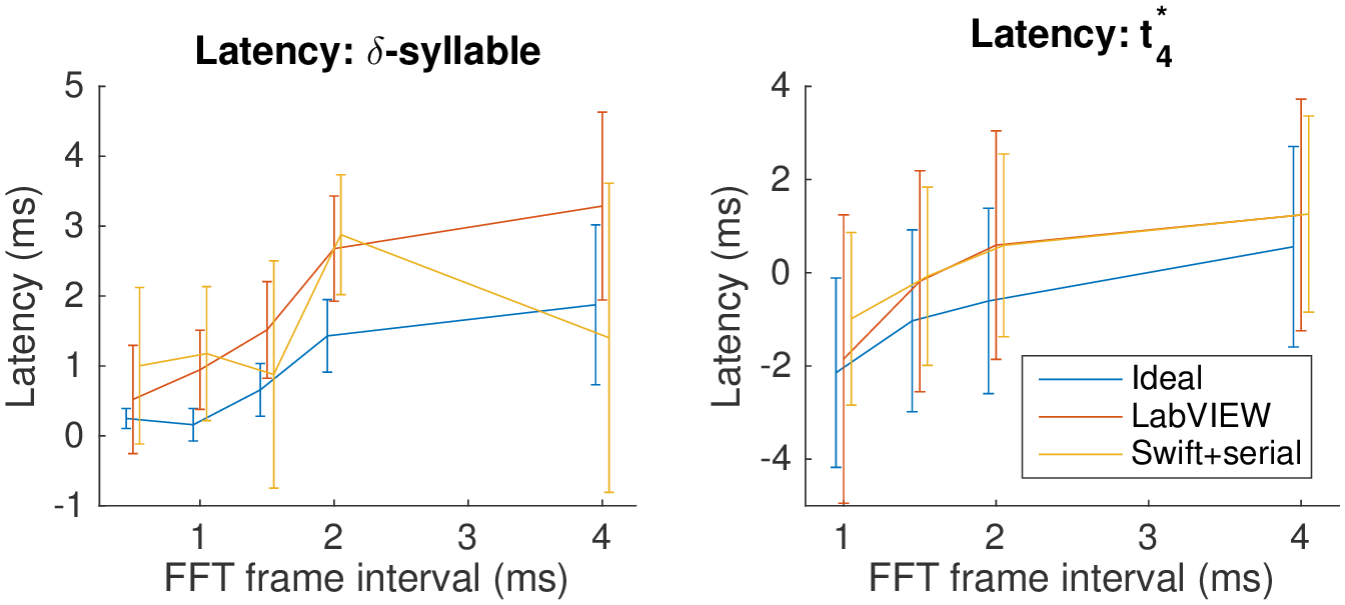
Timing varies as the FFT frame interval changes. Here we show results for the ideal detector and the LabVIEW and Swift+serial implementations, for the constructed *δ*-syllable and for trigger $t_{4}^{*}$ of lny64’s song. The lines show latency; error bars are standard deviation (jitter). Points have been shifted horizontally slightly for clarity; original positions are [0.5 1 1.5 2 4] ms.

Detector latency and jitter depend on the FFT frame rate. Our 1.5-ms–frame default is a compromise: shorter frames increase the precision of timing, but also increase computational requirements both during training and at runtime. Fig 4 shows how these numbers vary over a useful range of frame rates on our ideal detector, the Swift detector with serial output, and for the LabVIEW detector, for both the *δ*-syllable and for lny64’s song at $t_{4}^{*}$.

#### 3.2.2 Syllable choice

**Fig 5:**
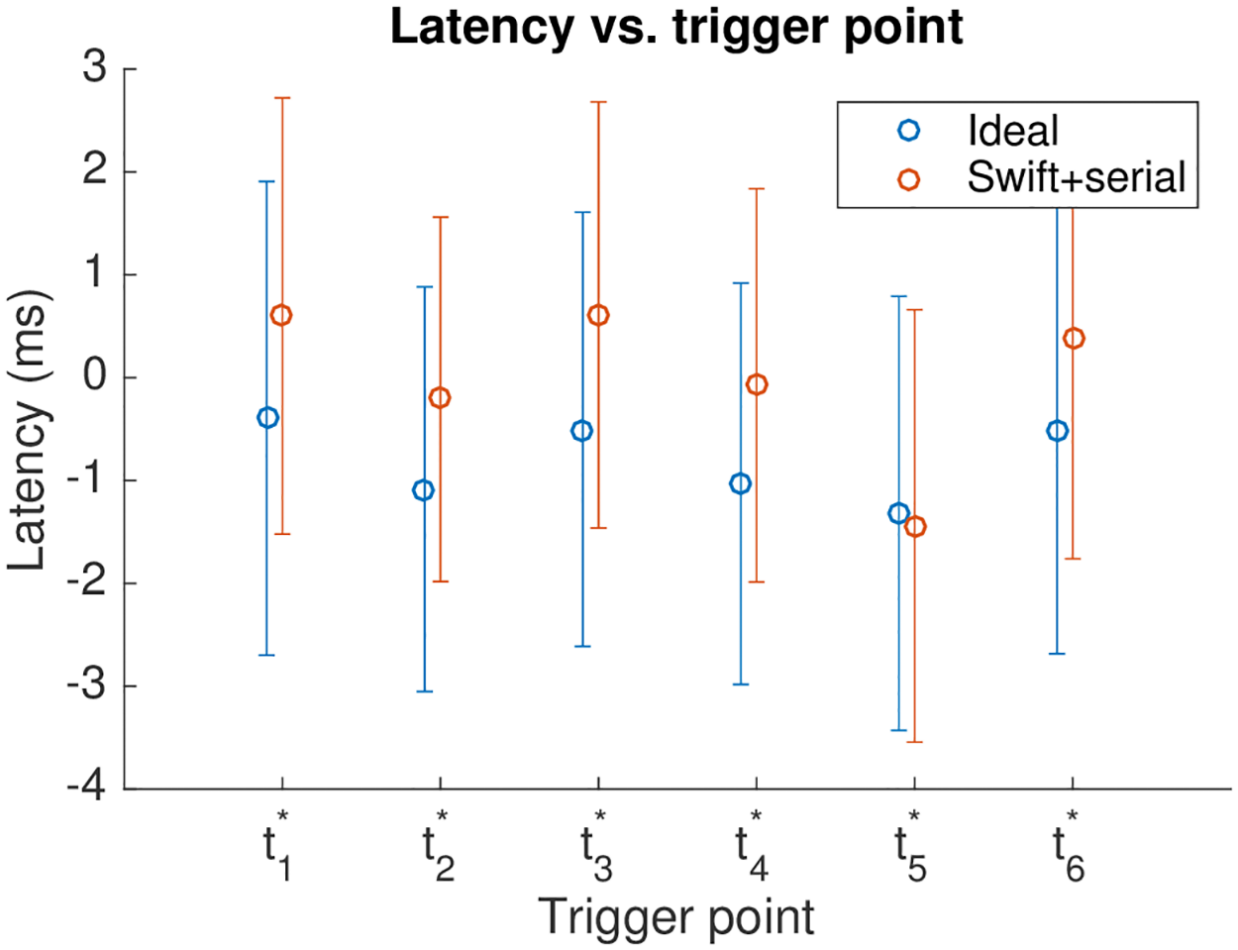
Timing data for lny64’s 6 test syllables, for the ideal and the Swift+serial detectors, with an FFT frame rate of 1.5 ms. Point centres show latency; error bars show jitter.

Syllable choice impacts detector performance, but despite the variety of syllables shown here, performance was stable across syllables. Fig 5 shows measured timing data for lny64’s test syllables for the Swift+serial detector compared to the ideal, with *t*_fft_ = 1.5 ms. Given the variety of the test syllables, the variability is surprisingly low, and is summarised in Table 3.

**Table 3:** Latency and jitter variability (95% confidence) for lny64’s six test syllables.

Detector	Latency	Jitter
Ideal	−0.8 ± 0.3 ms	2.1 ± 0.1 ms
Swift+serial	0.0 ± 0.6 ms	2.0 ± 0.1 ms

The negative latency is due to the way in which the network responds to the song through time: as the recognition region looks increasingly similar to the trained match, the network’s evaluation of similarity rises, and will generally cross the triggering threshold before it reaches its maximum value. A heuristic as simple as triggering at an apparent turning point after crossing the threshold might improve timing consistency at the expense of latency, but we did not test this.

#### 3.2.3 Detector implementations

**Fig 6:**
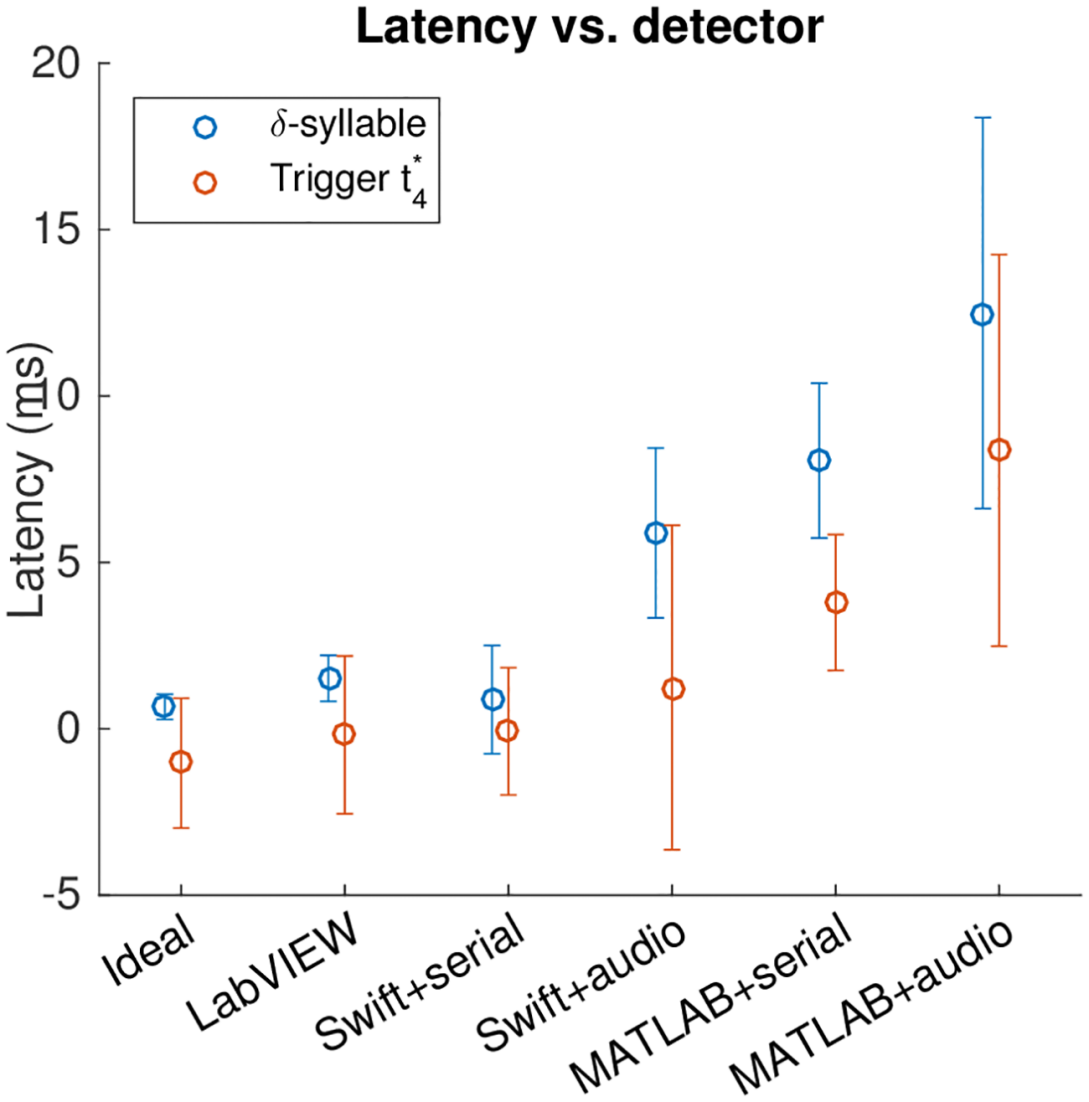
The different detectors for the constructed *δ*-syllable and for lny64’s song at $t_{4}^{*}$. Point centres show latency; error bars show jitter.

**Fig 7:**
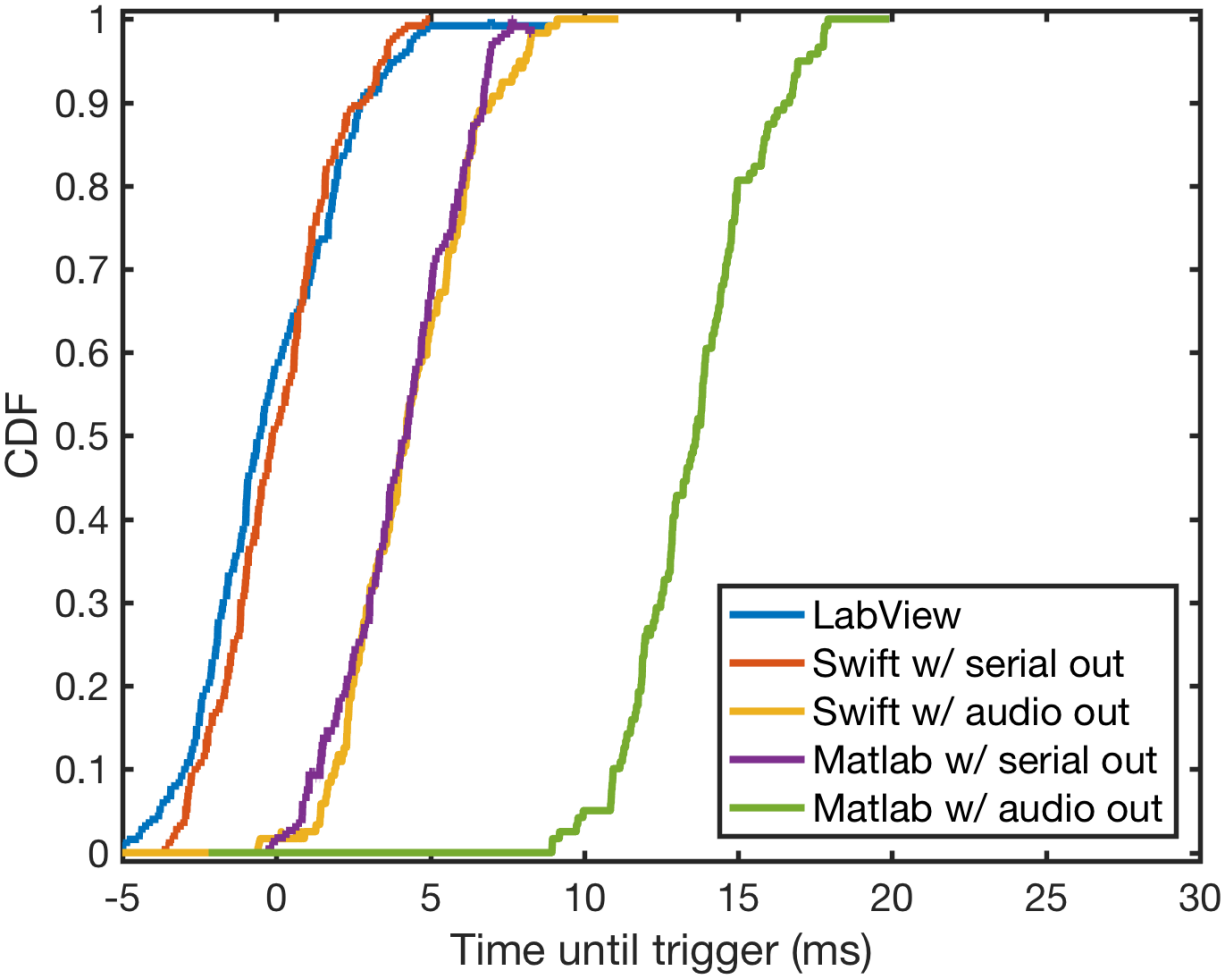
Raw timing curves for all detectors measured during detection of lny64’s $t_{4}^{*}$ using 1.5-ms frames. We extract the trigger events from each curve, from which we obtain the mean—latency—and standard deviation—jitter.

We compared latency and jitter across our different detector implementations for the *δ*-syllable and lny64’s $t_{4}^{*}$, again with *t*_fft_ = 1.5 ms. Results are shown in Table 4 and Fig 6. Fig 7 gives a more detailed view of what the timing curves look like for the five implementations of our detector on lny64’s $t_{4}^{*}$.

**Table 4:** Latency and jitter for each of our detector implementations on the synthetic *δ*-syllable and on syllable $t_{4}^{*}$ from lny64.

Detector	*δ*-syllable		lny64: $t_{4}^{*}$	
	Latency (ms)	Jitter (ms)	Latency (ms)	Jitter (ms)
Ideal	0.66	0.38	-1.0	2.0
LabVIEW	1.5	0.69	-0.18	2.4
Swift+serial	0.88	1.6	-0.075	2.0
Swift+audio	5.9	2.6	1.2	4.9
MATLAB+serial	8.1	2.3	3.8	2.0
MATLAB+audio	13	5.9	8.4	5.9

## 4 Discussion

This syllable detector is appropriate for zebra finch song, and although our tests were carried out on songs from that species, it is also likely to work well for Bengalese finches due to their similarly stereotyped syllables. It offers the following benefits:

- The detector is accurate. False negative and false positive rates can be well under 1% and 0.005% respectively, and trading these two numbers off against each other is through a single relative-cost parameter.
- Latency is generally under a millisecond, with jitter around 2 ms.
- Works on a wide range of target syllables using the default values described here, generally eliminating the need for hand-tuning.
- Runs fast enough for syllable-modification experiments on inexpensive consumer-grade hardware, although we recommend that the training phase be run on a fast desktop system with 32 GB of RAM.
- A single detector can generate different target pulses for multiple syllables at almost no additional computational cost during runtime, although training time will increase.

Although there are differences, the Swift+serial detector and the LabVIEW implementation are roughly comparable in performance. We prefer the Swift implementation due to its lower hardware and software requirements and the difficulty of debugging LabVIEW programmes. With serial output, MATLAB’s performance is good, although its buffer handling is sensitive to system load.

The songs presented here were recorded with fixed microphones mounted inside the cages. We found that higher accuracy is achieved when a microphone is instead mounted on the bird’s head, which maintains volume and reduces changes in timbre as the bird moves.

A common experimental paradigm requires detecting the frequency of syllables. Many pitch detection techniques rely on the spectrum, which incurs no additional computational cost here since it is already available. For example, [8] achieved good results with the Harmonic Product Spectrum algorithm [15].

In order to monitor syllable duration, the beginning and end of a syllable may be detected by looking at the ratio of total energy in the singing frequency band to the total energy, over some small time window. Any syllable thus identified that also contains a trigger event may be monitored for duration. Alternatively, the network can be trained to recognise both the beginning and the end of the syllable of interest.

In feedback experiments such as frequency- or duration-shifting, vocal output changes over the course of the experiment. The neural network excels at identifying syllables close to its training set, so as vocal output changes the detector may not recognise a match. If the detector must be robust to this shift, it may be retrained as often as necessary as the bird learns, or data consisting of synthetically pitch-shifted or duration-shifted target syllables over the recognition region may be added to the training set. We will test these approaches in future work.

## Supporting information

S1 Appendix A Online resources. Where to find the datasets and software introduced in this paper, with an overview of how to install and use the packages.
(PDF)
