# Supplementary material for: A fast and accurate zebra finch syllable detector
Source: PLoS One. 2017 Jul 28;12(7):e0181992. doi: 10.1371/journal.pone.0181992 (PMC5533338; doi:10.1371/journal.pone.0181992)
Supplement: S1 Appendix A — Where to find the datasets and software introduced in this paper, with an overview of how to install and use the packages. (PDF) [file pone.0181992.s001.pdf]

A Online Resources

A.1 Data

Data and training configuration files for the 8 birds discussed here are available from the Open Science Framework at DOI: 10.17605/OSF.IO/BX76R . DOIs for the versions of the programmes used here are provided for reproducibility; however, we generally recommend downloading the latest release.

A.2 Song alignment

Jeff Markowitz's song alignment software [11] can be found at
https://github.com/jmarkow/zftftb

A.3 Training the neural network

https://github.com/gardner-lab/syllable-detector-learn

DOI: 10.5281/zenodo.437555

Installation: See `README.md` in the repository for complete, up-to-date instructions.

- Clone the Github repository onto your computer, and add it to your MATLAB path.
- You also need the following packages from MATLAB File Exchange in your MATLAB path:

  Significant Figures by Teck Por Lam. File ID #10669.

  Generate maximally perceptually-distinct colors by Tim Holy. File ID #29702.

Usage:

- The main programme is `learn_detector.m`.
- The top section of `learn_detector.m` describes the configuration variables.
- The current directory should contain the data in `song.mat`. The format for this file is specified in `README.md`: a MATLAB file containing aligned song, nonsong (calls, cage noise, etc), and the audio sampling frequency.
- `learn_detector` also looks in the current directory for a configuration file `params.m`, described in `README.md`. If the file is empty, the programme will display an image showing the average of the song spectrograms (frequency vs. time), tell the user how to define syllables of interest, and exit. If `times_of_interest_ms` is defined, the programme will train the neural network. Optionally, `params.m` can also override any of the configuration parameters at the top of `learn_detector.m`.
- Configuration may also be controlled by specifying parameter-value pairs to the function call in the common MATLAB fashion; for example, `learn_detector('times_of_interest_ms', [150 200])`.

- The software will produce a neural network file, `detector_bird_times_....mat` (the ellipses signify other information encoded in the filename). It will also produce the audio test file described in Methods.

A.4 Runtime

A.4.1 MATLAB

https://github.com/gardner-lab/syllable-detector-matlab

DOI: 10.5281/zenodo.437557

Installation:

- Clone the Github repository onto your computer.
- Usage requires MATLAB with the Data Acquisition Toolbox and the Parallel Computing Toolbox.

Connection:

- Either connect the microphone directly into the microphone jack or through a preamp into the audio input jack. The current implementation only supports a single channel of audio.
- To generate TTL signals via the audio output, use the headphone or audio output jack.
- To generate TTL signals via an Arduino, switch to the `arduino` branch, load the MATLAB Arduino IO sketch onto the Arduino (the sketch is available in the Github repository, and provides a simple serial protocol for controlling pins). Connect the Arduino to the computer via USB. The TTL signal will be available on pin 7.

Usage:

- Switch to the directory of the repository and run `nndetector_live`. You can optionally specify parameters as string/value pairs. When you run the command, you will be prompted to select an input device, output device and select a ".mat" file containing the trained detector.
- Note that you may need to tune the input and output buffer based on computer performance. Try decreasing the size of the buffers until you see warnings about buffer under/overruns, then increase the value slightly. We usually found 2-3ms worked reliably. To specify the buffer sizes, use the `buffer_size_input` and `buffer_size_output` parameters.
- Information about the detector is logged to a file called "detector_status.log".

A.4.2 Swift

https://github.com/gardner-lab/syllable-detector-swift

DOI: 10.5281/zenodo.437559

Installation:

Download the most recent release from the Github repository onto your computer.

Connection:

- Either connect the microphone directly into the microphone jack or through a preamp into the audio input jack. If the input jack supports multiple channels (e.g., audio input jacks are usually stereo), you can connect multiple audio sources and run different detectors on each channel simultaneously.
- To generate TTL signals via the audio output, use the headphone jack. The audio output must have at least the same number of channels as the input.
- To generate TTL signals via an Arduino, load the MATLAB Arduino IO sketch onto the Arduino (the sketch is available in the Github repository, and provides a simple serial protocol for controlling pins). Connect the Arduino to the computer via USB. The TTL signal for the first input will be available on pin 7, the second input on pin 8, etc.

Usage:

- Use `convert_to_text.m` to convert the Matlab output file to a format easily readable by Swift. This will generate a text version of the neural network.
- Launch the application downloaded above. It will provide an menu to select an audio input and either an audio output or an Arduino output (if detected). Once you select an input and an output, a new window will show all audio channels associated with the input. For each channel, select the text-encoded version of the detector created in the previous step. Once configured, press run to begin monitoring the inputs.

Customisation:

- You can clone the Github repository and use Xcode to edit the project, if you are interested in extending or modifying the functionality. To hook into either the raw input or output, look at the "Processor.swift" file. The `Processor` class acts as a coordinator, receiving incoming audio, passing that to the detector and generating appropriate output TTL signals.

A.4.3 LabVIEW

https://github.com/gardner-lab/syllable-detector-runtime-labview

DOI: 10.5281/zenodo.437558

Installation:

Clone the Github repository onto your computer.

Connection:

- The microphone should be connected, optionally through a preamplifier, to the data acquisition card, by default on Analog Input 0 (AI0).
- Outputs are on the Digital I/O (DIO) channels. See the block diagram of `nnfft.vi` for documentation, or just connect to DIO0.

Usage:

- Use LabVIEW to open `nnfft.vi`.
- A file chooser is provided in the front panel of `nnfft.vi`. Use it to point to the network file produced by `learn_detector.m`, and use LabView's "Run" button to begin detection.
